# Special Issue “Endemic Arboviruses”

**DOI:** 10.3390/v14030645

**Published:** 2022-03-21

**Authors:** Didier Musso, Dominique Rousset, Christophe Peyrefitte

**Affiliations:** 1 Laboratoire Eurofins Labazur Guyane, 97300 Cayenne, French Guiana; 2 Aix Marseille University, IRD, AP-HM, SSA, VITROME, IHU-Méditerranée Infection, 13007 Marseille, France; 3 National Reference Centre for Arboviruses, Institut Pasteur de la Guyane, 97300 Cayenne, French Guiana; drousset@pasteur-cayenne.fr; 4 Institut Pasteur de la Guyane, 97300 Cayenne, French Guiana; cpeyrefitte@pasteur-cayenne.fr

Arthropod-borne viruses (Arbovirus) is an ecological term defining viruses that are maintained in nature through biological transmission between a susceptible vertebrate host and a hematophagous arthropod such as a mosquito. Most of these viruses cause zoonoses that usually depend on nonhuman animal species for maintenance in nature [1]. More than 500 arboviruses belong to the *Flaviviridae*, *Togaviridae*, and *Reoviridae* families or to the Bunyavirales order. They are registered in the International Catalogue of arthropod-borne viruses, of which more than 100 are implicated in human diseases.

In the past 40 years, arboviruses have emerged as a major global health problem with the resurgence of several well-known arboviruses, such as West Nile virus (WNV) [2], dengue virus (DENV) [3], or chikungunya virus (CHIKV) [4]. Others, such as Zika virus (ZIKV), emerged unexpectedly, thus causing major outbreaks associated with new clinical patterns (congenital Zika syndrome) and a new mode of transmission (sexual transmission) never before described for arboviruses [5].

As an introduction to this Special Issue, we would like to highlight with a few examples showing the unpredictability of arboviruses’ behavior and, consequently, that all arboviruses—whether or not they cause human diseases—and all arthropods that are potential vectors of arboviruses, should be monitored [6].

Over a 10-year period (1937 to 1947), the Yellow fever research institute in Uganda discovered seven new viruses (West Nile virus in 1937, Bwamba virus in 1937, Semliki forest virus in 1942, Bunyamvera virus in 1943, Ntaya virus in 1943, Uganda S virus in 1947, and Zika virus in 1947) [7]. WNV and ZIKV emerged on a large scale; what is the future of the other viruses? 

For unknown reasons, other arboviruses did not emerge in areas where all conditions are present for their emergence; for example, we do not know why the yellow fever that can be transmitted by *Aedes aegypti* mosquitos never emerged in South East Asia and Pacific regions where *Aedes aegypti* is responsible for major outbreaks of Dengue, Chikungunya, or Zika viruses [8]. Similarly, Mayaro virus (MAYV), Venezuelan equine encephalitis virus, or Oropouche virus have only been reported in South and Central America [9]. 

Some arboviruses have been reported in only one outbreak, such as the unexpected story of Rocio virus that was responsible for only one known outbreak in the 1970s in São Paulo state, Brazil, which lead to more than 1000 human encephalitis cases with a mortality rate of 10%. Subsequently, only a few sporadic infections have been reported [10].

In addition, arboviruses diseases are not a health problem limited to tropical and subtropical areas. The first explanation for this is that there is an increasing number of arboviruses infections from imported cases, as seen with Zika virus during the past pandemic [11] that are associated with non-arboviral local transmission (e.g., sexual and blood transfusion transmission) [5]. The second explanation is that arboviruses outbreaks may occur in temperate regions, as was demonstrated by the emergence of West Nile virus in New York City, USA, in 1999 in before spreading around the US [12]. 

As arboviruses are principally transmitted by mosquitoes, mosquito monitoring is a key point for surveilling arboviruses. However, countries without known arboviral vectors are not protected because the area of arbovirus vector mosquitoes is expanding, and arboviruses have the potential to adapt to new vectors and hosts—this was seen when DENV and ZIKV adapted from sylvatic mosquitoes and non-human primates to urban mosquitoes and humans [13].

This Special Issue contains five publications that illustrate arboviruses surveillance challenges.

Hansen et al. report cytokines profiles associated with long-term WNV neurological complications and chronic kidney disease sequelae.

Nebbak et al. report a virome study of three mosquito species, *Aedes albopictus*, *Culex pipiens*, and *Culiseta longiareolata*, which act as vectors of human infectious diseases in a small suburban city in the South of France using high throughput sequencing techniques.

Torres et al. describe the intra-host genetic diversity of dengue virus serotype 2 (DENV-2) that has been circulating in Brazil since 1990 and causes severe diseases and explosive outbreaks. They also correlate DENV-2 genetic diversity to its pathogenic potential and thus contribute to understanding the virus’s dynamics within its human host.

Bengue et al. evaluate the potential neurotropism of MAYV and show that human brain cells, particularly astrocytes and neural progenitors, are permissive to MAYV infection, which, consequently, could allow MAYV to invade the central nervous system and lead to MAYV-induced neuropathology. 

Golender et al. report identification and genetic characterization of viral pathogens, including arboviruses, in ruminant gestation abnormalities in Israel from 2015 to 2019.

We are grateful to the authors that contributed to this Special Issue.
